# Back to the future? Health and the World Bank’s human capital index

**DOI:** 10.1136/bmj.l5706

**Published:** 2019-11-13

**Authors:** Felix Stein, Devi Sridhar

**Affiliations:** Medical School, Edinburgh University, Edinburgh, UK

## Abstract

**Felix Stein** and **Devi Sridhar** warn of the dangers of subsuming health to economic productivity

Over the past 25 years, the World Bank has become one the world’s most influential global health institutions.^1^ ^2^ In October 2018 the human capital index (HCI), its latest major knowledge product, was launched. In this article we briefly describe what human capital is, before taking a closer look at what exactly the HCI measures. We then assess the index’s strengths and weaknesses for improving health worldwide. We argue that the HCI as an expression of human capital theory subsumes healthcare to the goals and logics of economic growth. While this makes it potentially relevant for neoclassical economists, it is of limited use for guiding healthcare policy overall.

## What is human capital?

Human capital can broadly be defined as all those economically productive aspects of human beings that are inseparable from their bodies. This is not a new idea. It is part of a long history of discussions among economists of how to bring about a workforce that is talented, disciplined, skilled, and healthy. Adam Smith was one of the most prominent figures to argue that people’s productive abilities should be seen as a form of capital, one that is fixed and realised within persons.^3^ That said, the term “human capital” started to become popular only from the late 1950s. At that time, Chicago School economists began using it systematically to discuss a series of labour related phenomena. These included income distribution inequalities^4^; macroeconomic growth^5^; as well as unemployment rates, workplace leadership styles, and workforce education.^6^ ^7^ The idea that health could be part of human capital gained traction in the early 1970s,^8^ and it has remained a staple of health economics textbooks.

Human capital was a political concept from the start. Developed during the height of the Cold War, it eliminated the notion of class from economic analyses by postulating that workers cannot be exploited by capital because they carry capital within themselves.^7^ ^9^ Thereby, the concept contributed to downplaying imbalances of power that arise as part of capitalist production. Today, human capital has made its way through business and economics departments into policy circles.

## The human capital index

The World Bank has long relied internally on the idea of human capital,^10^ ^11^ but only recently has it begun to promote it externally as an explicit guiding concept for governing health and education.^12^ It does this via the Human Capital Project, as part of which bank staff advocate the importance of human capital in public speeches and reports, do research on the foundations of the concept, and refer to it when working with client governments.^13^ A key element of this project is the HCI, which ranks countries according to how much human capital they are expected to generate with the goal of bringing about policy change.^14^ ^15^ The bank’s exact definition of human capital varies, as sometimes it refers to people’s economically relevant skills, knowledge, and health and at other times includes their “resilience.”^14^ In any case, the bank remains convinced that health is an important part of human capital, as people are generally more productive when they are healthier.

Looking at the HCI in detail, we see that it tries to do something quite remarkable. It estimates how much potential economic productivity may remain unrealised around the world owing to the poor health and modest education of the labour force. To achieve this, the index considers three features of people’s lives that are assumed to constrain their productivity. These are child mortality, insufficient education, and poor health. The three main components of the index are combined into a single number, known as the HCI score.

The score ranges from 0 to 1 and is meant to reflect how much people’s productivity deviates from an ideal state of 100% survival, perfect education, and perfect health. Thus, a score of 0.70 indicates that future workers born today will be on average 30% less productive than they would be at perfect survival, education, and health. Importantly, the HCI score is easily converted into potential gains in gross domestic product (GDP). Thus, a country with a score of 0.50 is predicted to be able to double its GDP if it reached the benchmark of complete education and full health.^14^

Let us consider the HCI’s three components in detail.^14^ ^16^

Component one measures child survival by using mortality rates of children under the age of 5 years based on data from the United Nations Interagency Group for Child Mortality Estimation. Since children who do not survive childhood will never become economically productive adults, their productivity estimates are reduced by a factor equal to their survival rate. Thus if child mortality is 5%, the bank reduces its productivity expectations for this component by 5%.

Component two measures education by keeping count of learning adjusted years of schooling. The bank takes stock of the years of formal schooling that children receive between the ages of 4 and 18 by looking at international school enrolment rates. To account for differences in the quality of education, years of schooling are adjusted, based on scores from international student achievement tests.

Component three is called the health component. In the absence of a standard measure for health of the workforce, it relies on two proxy measures. The first is adult survival, measured as the share of 15 year olds who survive until the age of 60, as reported by the UN Population Division. The second is the rate of stunting for children under 5, as reported by the Unicef-WHO-World Bank joint malnutrition estimates. The bank then assumes that a 10% increase in adult survival rates raises productivity by 6.5% and that a 10% reduction in stunting raises worker productivity by 3.5%, using correlations between height and income as a proxy.^16^

In summary, the HCI converts expected child survival, quality adjusted years of schooling, adult survival, and stunting rates into estimates of future worker productivity. The results are then combined via multiplication into a single HCI score that can easily be converted into expected shortcomings of income and GDP (table 1). The HCI thus subsumes health and education to economic concerns. This distinguishes it from other development indices, like the UN Development Programme’s human development index, which combines health, education, and economic productivity on an equal footing into a broader notion of development.^17^

**Table 1:** Measuring the productivity as a future worker of a child born in 2018 (maximum productivity=1)^14^

Human capital index component	
Component 1: survival	
Probability of survival to age 5	0.95
*Contribution to productivity*	*0.95*
Component 2: education	
Expected years of schooling	9.5
Test score (out of roughly 600)	375
Quality adjusted years of schooling	5.7
*Contribution to productivity*	*0.51*
Component 3: health	
Fraction of non-stunted children	0.68
Adult survival rate	0.79
*Contribution to productivity*	*0.88*
Overall human capital index	0.43

Overall productivity=survival based productivity×education based productivity×health based productivity.

## Strengths of the HCI

In subsuming health to economic concerns, the HCI serves those people who are particularly interested in the potential effects of policy interventions on GDP. It is designed to look for the sources of economic growth and to enable policy makers around the world to bring about such growth. It equally foregrounds the negative economic distributional effects of insufficient healthcare. This may influence policy makers interested in alleviating economic inequality to consider healthcare in their efforts. Taken together, the HCI further establishes the World Bank as a dominant institution for growth based development.

Since the HCI considers health spending a potentially profitable form of capital investment, it might bring greater importance to health considerations in overall governance debates. Indeed, the index has been explicitly designed to expand conversations about health from ministries of health to the more powerful ministries of finance and maybe even heads of government.^18^ This may tackle the distributional problem that healthcare tends to be underfunded in many developing countries. It may also dampen the bank’s past tendencies of cutting public health expenditure, limiting tax rates, reducing food sovereignty, and curbing worker protection.^19^ ^20^

Finally, the HCI already seems to “work” for the bank itself. It contributes to the bank’s aspirations to measure not just economic growth but also global economic wealth.^12^ It spurs further data gathering efforts around health as it relies on country data rather than data estimates.^21^ Moreover, human capital, which started as a concept for analysing labour, enables the bank to make sense of ongoing changes in the global job market.^22^ Lastly, the Human Capital Project may open up new markets for lending and advice, beyond the bank’s traditional focus on infrastructure investment.^13^ It has already gained the official support of over 57 countries,^23^ while non-government organisations have also been eager to adopt the term, even if some of them use it in a less economistic sense.^24^

## Weaknesses of the HCI

The HCI’s main disadvantages also stem from its origins in human capital theory, which subsumes healthcare to economic concerns. Doing so ignores decades of research from scholars who have tried to arrive at more holistic understandings of development, ones that the HCI does not reflect.^19^ ^25^ Three main issues arise within the field of healthcare.

Equity concerns are the first. As a foundation of health policy, human capital theory explicitly addresses the health issues of those people who may at some point become formally economically productive. This excludes anyone with disabilities that preclude participation in the labour market, people who are elderly or chronically ill, and those who are unwilling or otherwise unable to work. From the perspective of government, it also excludes those who may move outside of their jurisdiction. Moreover, human capital based healthcare systematically favours those workers who promise the highest income increments—often people who already are affluent or well educated. Within this privileged target population, the idea of human capital favours those illnesses that have a clearly negative effect on economic productivity.^16^ These conceptual shortcomings in terms of equity make human capital a highly questionable foundation for health policy design.

Secondly, the notion of human capital reconfigures the responsibility for health financing in uncertain ways. Since the idea of human capital arose, it has remained contested who should be “investing” in it. Should workers pay for their own healthcare since their human capital may one day raise their incomes? Are employers responsible for employee health, because it may boost company profits? Or should governments pay for healthcare because this may increase their stock of national wealth?^3^ ^12^ In recent years, the World Bank has argued the last, holding that national wealth can enable macroeconomic growth.^17^ ^21^ Yet, historically, the idea of human capital has been used to render individual workers responsible for their health, and it has extended this responsibility from on-the-job behaviour into their private lives.^8^ ^26^ ^27^ This is why it has been developed and supported by Chicago School scholars and why it remains so appealing to market libertarians today.

**Fig 1:**
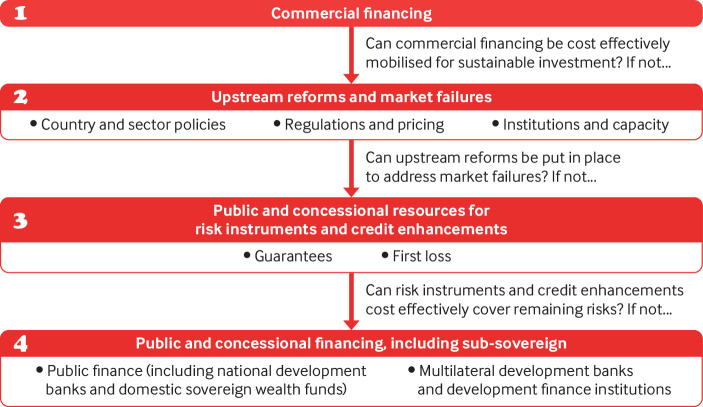
World Bank’s cascade approach^34^

Lastly, human capital lends itself to the development of new individualised debt instruments, as part of a wider financialisation of health.^28^ ^29^ This has already taken place in the field of education. Here, so called “human capital contracts,” originally conceived by Milton Friedman,^30^ have been suggested by the World Bank as a financing mechanism for university students.^31^ ^32^ Since students assume that attending university will increase their human capital (in other words, increase expected income), they could appeal to capital markets to invest in them and own some of their inalienable capital. Investors thereby become entitled to a percentage share of the students’ future income for a set number of years. Such personal debt obligations on the basis of assumed human capital increases are realistically conceivable in healthcare, both in healthcare education—where they are already being proposed^33^—and in treatment financing. This would be in line with the bank’s current approach to project financing, known as the cascade approach, which systematically privileges private over public finance (fig 1).

## Conclusion

Uncertainties remain around the future effects of the HCI. Yet, what is already clear is that as part of the World Bank’s Human Capital Project the HCI reconfigures how healthcare is understood. The notion of human capital subsumes healthcare to GDP growth. This makes health more interesting for growth based policy initiatives and it may raise the profile of health policy overall. It can also strengthen the World Bank’s own role in global health and development. However, it equally bypasses attempts at working towards a holistic notion of development, raises equity concerns in healthcare, risks individualising the responsibility for health financing, and opens the doors for further indebting healthcare practitioners and patients. The HCI may thus lead us back to a time where markets were held to be a panacea for health policy. In short, it risks taking us back to the future.

**Key messages**

The HCI and the underlying human capital theory consider human health only in terms of its economic effects
This may make healthcare more interesting for proponents of growth based development and raise its importance in policy circles
However, it has major limitations for guiding health policy, as it raises equity concerns and may enable further individualisation and financialisation of healthcare

## Notes

Contributors and sources: The authors have several years’ experience of analysing and publishing on global health and form part of a larger team at the Global Health Governance Programme studying the influence of the World Bank in global public health. This article relies on formal and informal interviews with World Bank staff, participant observation at two World Bank Spring Meetings and literature reviews.

Provenance and peer review: Commissioned; externally peer reviewed.

This article is part of a series commissioned by *The BMJ. *Open access fees for this article are paid for by the Wellcome Trust, which had no involvement in the commissioning, peer review, or decision to publish.
